# From bench to bedside: The role of cough hypersensitivity in chronic cough

**DOI:** 10.1002/ctm2.1343

**Published:** 2023-07-27

**Authors:** Matthew G. Drake, Lorcan P. McGarvey, Alyn H. Morice

**Affiliations:** ^1^ Division of Pulmonary and Critical Care Medicine, Department of Medicine Oregon Health and Science University Portland Oregon USA; ^2^ Wellcome‐Wolfson Institute for Experimental Medicine, School of Medicine Dentistry & Biomedical Science, Queen's University Belfast Belfast United Kingdom of Great Britain and Northern Ireland; ^3^ Respiratory Research Group Hull York Medical School University of Hull Cottingham UK

**Keywords:** antitussives, cough sensitization, refractory chronic cough, unexplained chronic cough

## Abstract

**Background:**

Chronic cough is a burdensome condition characterized by persistent cough lasting longer than 8 weeks. Chronic cough can significantly affect quality of life, physical function and productivity, with many people troubled with a cough that lasts for months or even years. People with chronic cough commonly report a persistent urge to cough with frequent bouts of coughing triggered by innocuous stimuli, which has led to the concept of cough hypersensitivity.

**Main body:**

Both central and peripheral neural pathways regulate cough, and although mechanisms driving development of cough hypersensitivity are not fully known, sensitization of these neural pathways contributes to excessive cough triggering in cough hypersensitivity. Effective therapies that control chronic cough are currently lacking. Recent therapeutic development has focused on several ion channels and receptors involved in peripheral activation of cough (e.g., transient receptor potential channels, P2 × 3 receptors and voltage‐gated sodium channels) or central cough processing (e.g., neurokinin‐1 [NK‐1] receptors and nicotinic acetylcholine receptors).

**Conclusion:**

These targeted therapies provide novel insights into mechanisms underlying cough hypersensitivity and may offer new treatment options for people with chronic cough. In this review, we explore preclinical and clinical studies that have improved our understanding of the mechanisms responsible for chronic cough and discuss the most promising targeted approaches to date, including trials of P2 × 3‐receptor antagonists and NK‐1–receptor antagonists.

## Graphical abstract

Chronic cough is a burdensome condition characterized by persistent cough lasting longer than 8 weeks. This review explores the mechanisms of chronic cough by drawing upon preclinical and clinical studies, including those highlighting the concept that cough‐reflex hypersensitivity may underlie chronic cough. The most promising targeted therapeutic approaches to date are considered.

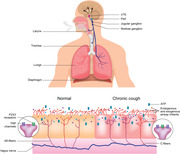

## 1 INTRODUCTION

Cough is one of the most common presenting complaints in primary care clinics.^1^ Although cough is a vital mechanism that protects the airways from mechanical and chemical irritants, for some individuals, cough can persist, becoming a chronic, pathologic condition that lasts for months or, in some cases, years.^2^ Chronic cough (defined as a cough lasting >8 weeks) has a worldwide prevalence of 4% to 5% and is often associated with pain, dizziness, urinary incontinence and even loss of consciousness.^3^, ^4^, ^5^ As a result, chronic cough contributes to considerable psychological stress, social stigma, reduced quality of life and impairments in activities of daily living and work productivity.^5^, ^6^ Moreover, owing in part to the ineffectiveness of existing cough‐suppressant therapies, people with cough often undergo repeated physician visits involving costly and extensive diagnostic workups and unsuccessful therapeutic trials.^3^, ^5^ Chronic cough is estimated to cost $6.8 billion and $156 million annually in the United States and Great Britain, respectively.^7^ Developing effective therapies, particularly those that specifically target pathologic chronic cough rather than globally suppressing all cough, remains a large unmet need.

People with chronic cough frequently report a persistent urge to cough and an enhanced sensitivity to stimuli, such as changes in ambient air temperature and exposure to aerosols and perfumes.^8^ In some instances, simply talking or singing provokes bouts of coughing.^9^ Chronic cough is a common feature in many conditions, including asthma, reflux disease and diseases affecting the upper airways (e.g., rhinitis and rhinosinusitis). Many people with chronic cough have no identifiable cause despite an extensive workup.^10^ These observations support the concept that chronic cough is a distinct clinical condition resulting from sensitization of neural pathways that evoke cough.^8^, ^11^, ^12^, ^13^

In this review, unless the cited literature provides a specific cause or subset of chronic cough, the term chronic cough will be used to indicate chronic cough due to comorbid conditions (e.g., asthma‐associated chronic cough and chronic obstructive pulmonary disease [COPD]), refractory chronic cough (RCC), or unexplained chronic cough (UCC). As hypersensitivity of the cough reflex is an underappreciated phenomenon and a relatively recently described concept,^11^, ^12^ we will review the current understanding of underlying mechanisms of cough hypersensitivity and the clinical presentation of this clinical population, as well as explore current treatment approaches and the future of antitussive therapies.

### 1.1 Clinical presentation and evaluation of cough hypersensitivity

The American College of Chest Physicians and European Respiratory Society have published guidelines for diagnosing and managing chronic cough.^7^, ^10^ The American College of Chest Physicians recommends systematically evaluating and treating common conditions that cause cough, including asthma, reflux disease, upper‐airway cough syndrome (UACS), nonasthmatic eosinophilic bronchitis and iatrogenic cough; ruling out cough‐inducing drugs such as angiotensin‐converting enzyme (ACE) inhibitors is also recommended.^14^ The European Respiratory Society characterizes chronic cough by various phenotypes of cough (i.e., asthmatic cough/eosinophilic bronchitis, reflux cough, rhinosinusitis and iatrogenic cough), though these guidelines do not refer to these conditions as causes of chronic cough.^10^ Both guidelines recommend using individual reports of the impact of cough, either through validated tools (e.g., Leicester Cough Questionnaire) or overall cough severity rating on a simple scale (e.g., “score your cough out of 10”).^10^, ^14^ Simpler scales are more likely to be used in clinical practice to assess clinical changes and response to treatment, whereas validated multidimensional tools are more frequently used to investigate efficacy of antitussives in clinical trials. Although these scales are not intended to facilitate diagnosis, they can be used to quantify cough burden and severity and may provide longitudinal information regarding the subjective experience of cough across visits.

People with chronic cough frequently present with symptoms suggestive of cough hypersensitivity, including a persistent or intermittent tickling sensation, irritation, rawness in the pharynx or laryngeal area and a choking sensation, all of which often cause an urge to cough or uncontrollable coughing.^8^ They may also report allotussia (cough triggered by typically innocuous physiological, mechanical, or chemical stimuli, such as talking, singing, exercise, or scents) or hypertussia (exaggerated or heightened cough response to tussive stimuli).^12^, ^13^, ^15^, ^16^ In a study of 60 people with RCC or UCC, all reported symptoms of cough hypersensitivity, with 95% also reporting abnormal laryngeal sensations (described as a tickle, globus sensation, irritation, dry throat, sore throat and presence of mucus in the throat) and over half reporting voice abnormalities.^16^

Parallels between the mechanisms of cough hypersensitivity and chronic pain have been reviewed and assessed elsewhere.^8^, ^17^, ^18^ Similar to allotussia and hypertussia observed in cough hypersensitivity, people with chronic pain report increased pain responses to innocuous stimuli (allodynia) and noxious stimuli (hyperalgesia) and central and peripheral sensitization resulting from inflammation is thought to drive these responses.^17^, ^19^ Like cough hypersensitivity, the mechanisms underlying hypersensitivity in chronic pain have been partially attributed to extracellular adenosine triphosphate (ATP) and P2X receptors, further emphasizing a role for extracellular ATP in hypersensitivity‐related conditions.^20^, ^21^

Although diagnostic tests and questionnaires have been developed for some conditions or aspects associated with chronic cough (e.g., Hull Airway Reflux Questionnaire for airway reflux symptoms associated with cough, oesophageal manometry for dysmotility and sinus imaging for UACS),^14^, ^22^ there are no formal diagnostic measures or objective biomarkers for cough hypersensitivity, and attempts to stratify individuals on the basis of sensitivity to common tussive triggers are limited given variability of cough responses in healthy individuals.^23^ For example, many studies use cough‐suppression and cough‐challenge tests, during which individuals are presented with a tussive stimulus (e.g., capsaicin) and instructed to either suppress cough (cough‐suppression test; evaluates central cough control) or cough as needed (cough‐challenge test; evaluates cough‐reflex sensitivity).^23^, ^24^ However, typical thresholds used in these tests to distinguish between normal and pathologic cough, including C2 and C5 (i.e., amounts of tussive stimulus needed to elicit 2 or 5 coughs, respectively), have been questioned on the basis of findings suggesting such thresholds display extensive overlap of health and disease and thus inaccurately reflect potential underlying cough sensitivity.^25^ Moreover, although more recent research has demonstrated that assessing the maximum number of coughs evoked by any concentration of a tussive stimulus may better discriminate pathologic from normal cough, this method may not be amenable to clinical practice owing to procedural complexities.^26^ Finally, clinical manoeuvers including those involving the Arnold nerve reflex (cough resulting from stimulation of the external auditory canal) and mechanical stimulation of points that may induce cough (cough resulting from pressure, flexion, or tension applied to somatic points in the upper trunk or neck) have been investigated, with people with chronic cough generally showing heightened cough responses with these manoeuvers when compared with healthy volunteers.^27^, ^28^, ^29^, ^30^ Although these tests can be useful for confirming vagal hypersensitivity, they are not widely used in clinical practice. Therefore, the development of sensitive tests or biomarkers for diagnosing cough hypersensitivity remains an important research aim.

### 1.2 Mechanisms underlying cough and cough hypersensitivity

**FIGURE 1:**
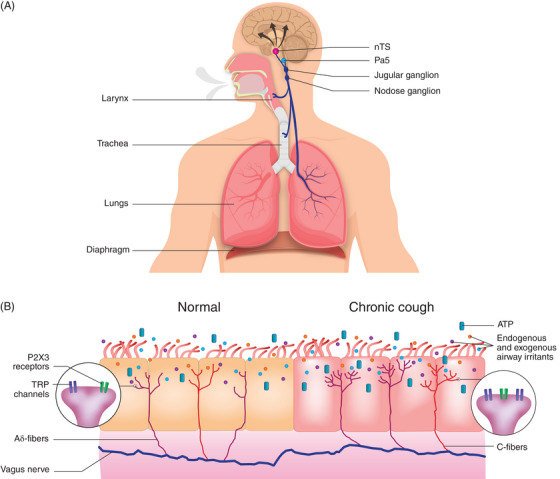
Central and peripheral cough mechanisms. (A) Cough is triggered by mechanical or chemical stimuli that activate peripheral receptors on airway sensory nerves (Aδ‐ and C‐fibres originating from jugular or nodose ganglia). Signals from the periphery are then relayed via the vagus nerve to the paratrigeminal nucleus and the nucleus of the solitary tract located in the medulla oblongata. Cough is supported by a distributed central network, including the thalamus, midcingulate cortex, insula, primary somatosensory and motor cortices, posterior parietal cortex, orbitofrontal cortex and cerebellum. Central cough centres send signals via efferent nerves in vagus, phrenic and spinal motor nerves to activate the diaphragm and muscles involved in cough. Substance P signaling via NK‐1 receptors in the CNS may be involved in inducing and maintaining cough hypersensitivity. (B) Endogenous and exogenous airway irritants and ATP trigger cough receptors, including TRP channels and P2 × 3 receptors, in both normal and chronic cough. Individuals with chronic cough are most likely exposed to more endogenous irritants versus healthy controls. Several changes in the peripheral airway have been proposed to underlie chronic cough, including inflammation and ATP release that may trigger cough, as well as an increase in nerve fibre length and branching within the airway epithelial layer. Although TRPV1 receptors appear to be upregulated in chronic cough versus healthy controls, the extent to which P2 ×3‐receptor upregulation occurs is not known. ATP, adenosine triphosphate; CNS, central nervous system; NK‐1, neurokinin‐1 receptor; nTS, nucleus of the solitary tract; Pa5, paratrigeminal nucleus; TRP, transient receptor potential; TRPV1, TRP vanilloid‐1.

Cough hypersensitivity may occur when there are changes to central or peripheral neuronal pathways that generate cough.^13^, ^31^ Several reviews thoroughly discuss anatomic mechanisms of cough.^32^, ^33^ Mechanical or chemical stimuli activate peripheral receptors on airway sensory nerves, triggering involuntary cough; subtypes of sensory nerves, termed Aδ‐fibres and C‐fibres, are specifically activated by these respective stimuli^8^, ^34^, ^35^ and relay signals to the paratrigeminal nucleus (Pa5) and the nucleus of the solitary tract (nTS) in the brainstem.^34^, ^35^ Neuroimaging has shown that in addition to the brainstem, cough in response to a tussive stimulus is supported by a distributed network, including the thalamus, midcingulate cortex, insula, primary somatosensory and motor cortices, posterior parietal cortex, orbitofrontal cortex and cerebellum.^18^ Cough may also be a conscious behaviour, either due to perception of the urge to cough or an entirely volitional response.^36^ Voluntary cough is processed cortically in the premotor and motor cortices, as well as in the cerebellum.^33^, ^36^ For both involuntary and voluntary cough, cough centres trigger efferent nerves in the vagus, phrenic and spinal motor nerves to coordinate activation of the muscles involved in cough.^34^ The mechanisms of cough hypersensitivity are illustrated in Figure 1.^8^, ^13^, ^18^, ^31^, ^32^, ^33^, ^34^, ^35^, ^37^, ^38^, ^39^

Alterations in cortical pathways involved in voluntary cough suppression may also contribute to the development of cough hypersensitivity. A neuroimaging study including people with cough hypersensitivity and healthy volunteers found that people with cough hypersensitivity not only exhibited increased activation in left, right and dorsal midbrain regions after capsaicin inhalation but also exhibited decreased activation in regions associated with cough suppression (i.e., dorsomedial prefrontal cortex and anterior midcingulate cortex).^18^ On the basis of these findings, the authors concluded that central mechanisms contribute to cough hypersensitivity through alterations in sensory processing and reduced capacity for cough suppression, leading to excessive coughing.^18^ A behavioural study of healthy volunteers and individuals with RCC provides additional support for loss of inhibitory cough control as a distinguishing feature of RCC and cough hypersensitivity.^24^ In that study, individuals were exposed to capsaicin inhalation with specific instructions not to cough during the test (i.e., cough‐suppression test); those with RCC were less able to suppress capsaicin‐evoked cough compared with healthy volunteers. Another study assessing the effect of pain conditioning on experimentally evoked cough used the application of a noxious stimulus (cold‐water immersion of the hand) to evaluate endogenous inhibitory cough mechanisms in individuals with RCC and healthy controls during a capsaicin inhalational challenge.^40^ The conditioned pain modulation paradigm has been used in studies of chronic pain to evaluate endogenous pain inhibition and was hypothesized to inform whether there are similar endogenous inhibitory mechanisms that control cough. Although individuals with RCC and healthy controls demonstrated similar capsaicin‐evoked urge‐to‐cough reductions during cold‐water immersion, those with RCC coughed more frequently than healthy controls despite reporting a similar urge to cough. The authors suggest that these results support that exposure to a noxious stimulus can modulate airway sensations and the cough reflex, but that those with RCC may have an impairment in this endogenous inhibitory control mechanism, potentially located within the periaqueductal grey, rostral ventromedial medulla, nTS and Pa5. Taken together, these studies collectively show that cough sensitivity may occur because of heightened responses of protussive pathways and/or loss of suppressive pathways in the cough reflex.

Most evidence regarding the aetiology of cough hypersensitivity has been generated from preclinical studies showing that exposure to airway irritants provokes airway inflammation and alters peripheral sensory or central nerve function. For example, in guinea pigs, interferon‐γ, which is released in response to viral infections, increases cough sensitivity to inhaled tussive stimuli by depolarizing the membrane potential of vagal sensory neurons.^41^ Murine models have additionally shown that tumour necrosis factor α increases sensitivity of peripheral airway sensory nerves.^42^, ^43^ Such alterations may, over time, sensitize peripheral cough receptors, resulting in increased activation by airway stimuli and excessive cough triggering. In the lower airways of humans with chronic cough, airway epithelial sensory nerve density is also increased relative to healthy volunteers, suggesting nerve plasticity involves changes in both morphology and function in the setting of chronic cough.^31^

A role for autacoids in sensitizing the cough reflex in response to other chemical stimuli has been proposed.^44^ Guinea pigs exposed to aerosolized bradykinin exhibited paroxysmal cough, with subthreshold bradykinin challenges inducing cough sensitization to higher bradykinin concentrations.^45^ In other studies, prostaglandin E_2_ significantly enhanced citric acid–induced cough in a conscious guinea pig model and led to membrane hyperpolarization of nodose and pulmonary C‐fibres to chemical and electrical stimulation in anesthetized rats.^46^, ^47^ Prostaglandin E_2_ enhancement of citric acid–induced cough may be driven by central enhancement of cough through E prostanoid receptor 3–dependent activation of voltage‐gated sodium channel 1.8 (NaV 1.8).^47^ Furthermore, it has been suggested that ATP is an important “priming autacoid” in the airways: ATP primes sensory neurons to have lowered activation thresholds to stimuli that drive afferent excitatory traffic, thus contributing to sensitization.^48^ For example, histamine may increase cough‐reflex sensitivity when released in the presence of ATP in the airways.^49^ This was shown in guinea pigs, where there was a significant increase in citric acid–induced coughs when pretreated with histamine.^49^

Considerable evidence garnered from inhalational challenges supports the concept of cough hypersensitivity. Compared with healthy volunteers, people with chronic cough exhibit heightened responses to a wide range of tussive stimuli, including capsaicin, citric acid, mannitol and hypertonic saline.^50^, ^51^, ^52^ In humans, there is a sex‐related difference in cough hypersensitivity. Women, both with and without chronic cough, have a heightened cough sensitivity, which appears to have an onset at puberty and is most likely due to enhanced central responses in the somatosensory cortex.^53^, ^54^ This may explain the overrepresentation of women seen in specialist cough clinics (e.g., 2:1 ratio of women to men) and supports observational studies showing a higher prevalence of chronic cough (which increases with age) in women relative to men (e.g., 5.2% vs. 4.7%).^5^, ^54^ However, the relative contributions of central versus peripheral vagal hypersensitivity in chronic cough are unclear. A double‐blind, randomized, two‐period, crossover study exploring the effect of a peripherally acting P2 X3 receptor antagonist, gefapixant, on four inhaled cough‐challenge agents found no significant effect of gefapixant in capsaicin or citric acid challenges, whereas gefapixant significantly inhibited cough in both ATP and distilled water challenges.^55^ Thus, together with results from phase 3 trials,^56^ gefapixant preserved irritant‐driven and protective cough and inhibited pathologic cough. These results suggest that pathways underlying irritant‐induced cough may be different from those underlying the pathologic hypertussia of chronic cough and support the use of more targeted antitussives to regulate pathologic cough responses while preserving the protective cough reflex.

Neuronally expressed ion channels and receptors are implicated in the development of cough hypersensitivity, including those involved in peripheral stimulation of cough (e.g., transient receptor potential [TRP] ankyrin‐1 [TRPA1], TRP villanoid‐1 [TRPV1], TRPV4, TRP melastatin‐8 [TRPM8], P2 × 3 receptors and NaV 1.7 and 1.8) and those involved in the transmission of signals in the central nervous system (CNS; e.g., neurokinin‐1 [NK‐1] receptors, nicotinic acetylcholine receptors).^33^, ^57^, ^58^, ^59^, ^60^ The TRPA1 and TRPV1 channels regulate tussive responses in murine and guinea pig models,^61^ and sodium channel blockers reduce capsaicin‐invoked cough in guinea pigs,^62^ thus demonstrating these peripheral channels play a role in irritant‐induced cough. Additionally, preclinical studies have shown that central administration of nicotine reduces cough in cats, and the α7 nicotinic‐receptor agonist ATA‐101 dose dependently inhibits cough in guinea pigs.^63^, ^64^ However, TRP channel antagonists, sodium channel blockers and nicotinic‐receptor agonists (i.e., ATA‐101) have failed to improve chronic cough in clinical trials.^65^, ^66^, ^67^, ^68^, ^69^ These findings suggest that the complex processes underlying chronic cough in humans are not solely explained by a single peripheral receptor or channel and that species‐specific differences in cough triggering may confound the translation of preclinical research into chronic cough therapies for humans.

Extracellular ATP may also play a prominent role in cough hypersensitivity. During cellular injury or inflammation, cells release ATP to alert neighbouring cells to damage.^48^ Excess extracellular ATP release may assist in sensitizing peripheral afferent neurons, leading to increased neural activation and cough triggering.^48^ Extracellular ATP activates several ionotropic P2X and metabotropic P2Y purinoceptor‐receptor subtypes, including homomeric P2 X3 and heteromeric P2 X2/3 receptors expressed by airway sensory neurons that mediate cough.^70^ Preclinical models have shown that both P2 X2/3 and P2 X3 receptors have a role in signalling during airway inflammation and injury,^71^ and that activation of P2X receptors by extracellular ATP results in increased cough sensitivity to capsaicin.^72^

In respiratory conditions associated with cough and airway inflammation, such as COPD and asthma, extracellular ATP may be elevated and sensitivity to ATP is heightened.^73^, ^74^ For example, a study comparing people with COPD, smokers and healthy nonsmokers showed that inhalation of aerosolized ATP provoked a more pronounced cough, reduced forced expiratory volume in 1 s (FEV_1_) by ≥20% and increased perceptions of dyspnoea in people with COPD and smokers compared with healthy volunteers. Aerosolized ATP provoked cough in 90% of all participants, with 81% reporting throat irritation and 29% reporting sputum production.^73^ In a similar study, aerosolized ATP increased cough, reduced FEV_1_ and increased perceptions of dyspnoea in people with asthma compared with healthy nonsmokers.^74^ Extracellular ATP is also elevated in bronchoalveolar lavage fluid samples from people with COPD following acute smoke exposure and people with asthma following an allergen inhalational challenge.^75^, ^76^ Similarly, a cohort of people with idiopathic cough had significantly increased cough sensitivity to inhaled ATP relative to healthy volunteers.^77^ Taken together, these findings support that in airway diseases, ATP has a mechanistic role in cough. Although much remains to be known regarding the production and metabolism of ATP in individuals with chronic cough, it is most likely produced within the epithelium or other cells (e.g., macrophages).^78^

The NK‐1 receptor and its ligand substance P may also be involved in inducing and maintaining cough hypersensitivity, both peripherally and centrally, either by indirectly mediating inflammatory mediators or directly stimulating sensory nerve fibres.^79^, ^80^, ^81^ In guinea pig models, substance P increased neuronally mediated airway responsiveness to histamine—potentially indirectly as a result of airway inflammation in response to sensory activation.^80^, ^82^ In another model, exposing guinea pigs to an aerosol of exogenous substance P significantly increased cough counts, whereas antagonizing substance P reduced cough counts in guinea pigs exposed to exogenous substance P.^83^ Clinically, substance P is elevated in nasal secretions of individuals with increased cough sensitivity^84^ and chronic cough.^85^ In a study of 10 people with idiopathic pulmonary fibrosis and 10 healthy volunteers, substance P did not elicit cough response in healthy volunteers but elicited a cough response in 70% of those with idiopathic pulmonary fibrosis.^86^ ACE inhibitors increase cough‐reflex sensitivity in humans, and accumulation of substance P, a substrate of ACE, has been hypothesized as the mechanism of ACE inhibitor–induced cough.^87^ These results support the role of airway inflammatory processes and substance P in eliciting cough.

Cough hypersensitivity is mediated by both peripheral and central mechanisms, and receptors for ATP and substance P (i.e., P2 X3 receptors and NK‐1 receptors, respectively) are emerging as important potential mechanisms for targeted antitussive treatment.

### 1.3 Rethinking the use of broad cough suppressants

When an underlying cause of chronic cough cannot be identified, and particularly when specific treatments for underlying conditions (such as reflux disease, asthma, or rhinosinusitis) are ineffective, clinicians commonly rely on therapies that globally suppress cough.^88^ Most commonly, opioid‐receptor agonists are employed,^89^ which may provide relief of cough in some situations but are associated with side effects (e.g., constipation and drowsiness),^88^ have potential for dependency, and may cause an unwanted suppression of protective cough reflexes.^90^ Cough‐suppression therapy delivered by a speech‐language pathologist has also demonstrated efficacy in improving cough^91^, ^92^; however, this treatment may not be widely accessible.^93^

Current guidelines also recommend the use of neuromodulators (e.g., gabapentin and pregabalin) to suppress cough, as these drugs are thought to target central sensitization by blocking central voltage‐gated calcium channels that may drive cough.^10^, ^94^ Some studies have shown benefit with neuromodulators, but, once again, adverse effects (i.e., dizziness, drowsiness and ataxia) limit their utility.^88^, ^95^ Although neuromodulators occasionally provide subjective benefit, they infrequently improve objective cough frequency, suggesting their efficacy is not driven by direct antitussive activity.^96^

Recent studies have suggested more targeted treatments have potential for addressing central and peripheral dysregulation of cough reflexes. In theory, these targeted agents may act specifically to reduce aberrant cough hypersensitivity rather than broadly suppressing cough. Drugs in development that target these mechanisms are summarized in Table 1. Although some approaches (e.g., TRPV1, TRPA1, TRPV4 channel inhibitors; Nav1.7 inhibitors) have failed to improve cough versus placebo in clinical trials to date,^65^, ^66^, ^67^, ^68^ others, such as P2 X3 receptor antagonists (supported by the role of ATP in airway inflammation and cough hypersensitivity) and NK‐1 receptor antagonists (potentially via targeting central dysregulation), have had more promising results.^96^

**TABLE 1:** Drugs investigated for the treatment of chronic cough.

Drug (manufacturer)	Mechanism of action	Development phase
Gefapixant (Merck & Co., Inc., Rahway, NJ, USA)	P2 X3 receptor antagonist	Phase 3 completed
		(NCT03449134, NCT03449147)
		Phase 3b completed
		(recent‐onset chronic cough; NCT04193202)
		Phase 3b completed
		(women with SUI and chronic cough; NCT04193176)
		Approved in Japan and Switzerland
Eliapixant (Bayer)	P2 X3 receptor antagonist	Phase 2 completed (NCT04562155)
		*Development discontinued*
Filapixant (Bayer)	P2 X3 receptor antagonist	Phase 1/2 completed
		(NCT03535168)
		*Development discontinued*
Camlipixant (Bellus)	P2 X3 receptor antagonist	Phase 2 completed (NCT04678206)
Sivopixant (Shionogi)	P2 X3 receptor antagonist	Phase 2 completed (NCT04110054)
NOC‐100 (Nocion)^†^	NaV 1.7 blocker	Phase 2a ongoing
		(EudraCT 2020‐004715‐27)
Orvepitant (NeRRe Therapeutics Ltd.)	NK‐1 receptor antagonist	Phase 2 completed (NCT02993822)
AX‐8 (Axalbion)	TRPM8 agonist	Phase 2 in progress (NCT04866563)

Abbreviations: NaV, voltage‐gated sodium channel; NK‐1, neurokinin‐1; SUI, stress urinary incontinence; TRPM, transient receptor potential melastatin.

^†^Additionally being pursued for acute cough.

Several P2 X3 receptor antagonists have been tested in clinical trials, including gefapixant, camlipixant, sivopixant, eliapixant and filapixant. Gefapixant is the most clinically advanced P2 X3 receptor antagonist, with data from two large, phase 3, randomized trials and a phase 3b randomized trial demonstrating significantly improved objective cough frequency and cough‐specific quality of life in people with RCC or UCC.^56^, ^97^ Gefapixant is currently approved for treatment of RCC or UCC in Japan and Switzerland, and approval is being sought in the European Union and United States.^98^ Camlipixant initially failed to meet its primary endpoint in a phase 2a study including people with RCC or UCC, though this study was terminated early because of the COVID‐19 pandemic.^99^ A subsequent phase 2b study of camlipixant enrolling an enriched population based on baseline cough frequency met the primary endpoint of significant reduction in 24‐h cough frequency.^100^ Another agent, sivopixant, did not meet its primary endpoint in a phase 2b study,^101^ and despite results showing efficacy, the eliapixant and filapixant clinical development programs have been discontinued.^102^, ^103^, ^104^, ^105^, ^106^

Although P2 X3 receptor antagonists have generally favourable safety profiles, taste‐related adverse events (e.g., hypogeusia and dysgeusia) are observed^106^, ^107^; this is most likely because the P2X receptors on airway sensory nerves are also expressed on the gustatory nerves innervating taste buds. After exposure to tastants, ATP is released from taste cells and subsequently activates P2X receptors, which then initiate action potentials to convey taste signals to the brain. Accordingly, antagonism of P2X receptors may affect taste. Although more selective P2 X3 antagonists have generally exhibited lower incidences of taste‐related adverse events in trials, results from studies of eliapixant and filapixant have suggested that receptor selectivity of P2 X3 over other P2X receptors may not completely explain taste‐related side effects, and other factors, including pharmacokinetic characteristics or allosteric binding sites, may be contributing to observed effects.^104^, ^108^ These taste effects typically occur within a few days of drug initiation and resolve when therapy is stopped.^56^, ^106^ Of note, many studies have consistently observed a large placebo response, though the mechanisms underlying this large response are not well understood.^56^, ^107^, ^109^ Reasons for differences in clinical efficacy between P2 X3 antagonists in clinical trials are also unclear but could be due to drug characteristics (e.g., receptor selectivity, pharmacokinetics, binding site), placebo effects, or individual factors (e.g., selection for baseline cough frequency). Additional data from phase 3 studies and head‐to‐head studies are needed to better understand the comparative efficacy and safety profiles of these agents.

The NK‐1 receptor antagonist orvepitant, which targets both central and peripheral cough mechanisms (unlike previous NK‐1 antagonists in clinical trials), was investigated in an uncontrolled phase 2 pilot study of RCC. In this study, 8 weeks of treatment significantly improved daytime and nighttime cough frequency from baseline and led to significant improvements in subjective measures of quality of life and daytime and nighttime cough severity.^79^ A subsequent phase 2b study similarly showed significant improvement in patient‐reported outcomes of cough frequency, severity and quality of life, although this latter study failed to show statistically significant improvement in objective cough frequency.^110^ Additionally, the NK‐1–receptor antagonist aprepitant has shown efficacy in reducing cough frequency and severity and improving cough‐specific quality of life in a randomized, placebo‐controlled trial of individuals with chronic cough due to lung cancer.^111^

Overall, studies of P2 X3‐receptor antagonists and the NK‐1 receptor antagonist orvepitant support the role of these receptors in chronic cough, as evidenced by the impact of these treatments on objective cough frequency or subjective patient experiences related to their cough. The generally positive outcomes of these studies contrast with previously reported negative clinical findings using inhibitors of irritant receptors such as TRPV1. These collective data illustrate the complexity in the neuronal mechanisms underlying chronic cough.

## 2 FUTURE DIRECTIONS

The studies reviewed above highlight the need for additional research to further understand the mechanisms of cough hypersensitivity and optimal treatment approaches. Much of our understanding of central mechanisms of cough comes from animal models, with human studies having been primarily conducted using functional magnetic resonance imaging.^18^ Improved methods for understanding neuronal expression, function and synaptic transmission in brainstem and cortical centres are needed to determine translational potential of previous research findings from animal models.

Future antitussive clinical trials should consider the following aspects to provide more information on mechanistic causes of cough hypersensitivity in humans and better characterize the chronic cough population. First, whether airway sensitization is reversible with targeted treatment of specific pathways remains to be established. Second, the source of the large placebo response in cough trials requires additional exploration. Third, nonpharmacologic interventions may provide relief for some individuals, but clinicians lack guidelines for determining which patients may benefit from nonpharmacologic versus pharmacologic approaches.^93^ Fourth, the observation that not all individuals respond to investigational therapies for chronic cough in clinical trials suggests that mechanisms driving cough are heterogeneous and may implicate distinct neurologic phenotypes. For example, further characterization of people with chronic cough who do versus do not respond to investigational P2 X3 or NK‐1 receptor antagonists may illuminate potential drivers of differential responsiveness of an individual's cough to different therapeutic mechanisms. Indeed, clinical study results have shown that individuals with different underlying causes of chronic cough (e.g., asthma, COPD and RCC) have disease‐specific changes in airway sensory nerve function, supporting evidence for different cough phenotypes.^112^ Ideally, further research will facilitate the identification of biomarkers that can aid phenotyping and support personalized therapy, as well as potentially inform the development of targeted antitussives.^94^ Finally, once targeted therapies become approved and widely available for chronic cough, ensuring the right individuals have access to these treatments will be important. Further research is needed to characterize mechanisms of cough hypersensitivity and drivers of cough to inform the future development of effective targets for pharmacologic intervention.

## 3 CONCLUSIONS

Chronic cough is a distinct clinical entity often accompanied by symptoms reflecting cough hypersensitivity, such as a chronic urge to cough and excessive cough in response to innocuous stimuli. Current evidence suggests cough hypersensitivity is driven by alterations in central and peripheral neural pathways, including sensitization of peripheral sensory nerves in airways and dysfunction of the central pathways that regulate cough triggering and cough suppression. Several compounds targeting neuronal channels and receptors have been investigated, with P2 X3 and NK‐1 receptor antagonists showing the most promise in clinical trials to date. Targeted treatments of chronic cough, particularly those that suppress pathologic cough without affecting the protective cough reflex, remain a large clinical need. Further research is needed to characterize mechanisms of cough hypersensitivity and drivers of cough in people with chronic cough, as this will inform the future development of effective targets for pharmacologic intervention.

## CONFLICT OF INTEREST STATEMENT

Matthew G. Drake reports receiving consultancy fees from AstraZeneca, Chiesi, GSK and MSD. Lorcan P. McGarvey reports receiving consultancy fees from Applied Clinical Intelligence, Bayer, Bellus Health, Bionorica, Chiesi, MSD, Nocion Therapeutics and Shionogi; receiving an investigator‐initiated grant from MSD; participating on a data safety‐monitoring/advisory board for AstraZeneca, Bayer, Bellus Health, Chiesi, MSD, Nocion Therapeutics, Shionogi and Trevi; and serving as cochair of ERS NEuroCOUGH Clinical Research Consortium. Alyn H. Morice reports receiving consultancy fees from Bayer, Bellus Health, Boehringer Ingelheim, MSD, Pfizer, Procter & Gamble and Shionogi; receiving payment or honoraria for lectures, presentations, or speakers bureaus from AstraZeneca, Boehringer Ingelheim and MSD; receiving grant support from Afferent, Infirst, MSD and Procter & Gamble; and serving as chair of the Hull University Teaching Hospitals Drug and Therapeutics Committee.
